# Effects of HyaRegen gel on tumour proliferation of colorectal peritoneal metastases

**DOI:** 10.1371/journal.pone.0307965

**Published:** 2024-09-10

**Authors:** Marie-Laure Perrin, Clément Bassetti, Sylvaine Durand Fontanier, Catherine Yardin, Sylvia M. Bardet, Abdelkader Taibi

**Affiliations:** 1 University Limoges, CNRS, XLIM, UMR 7252, Limoges, France; 2 Visceral Surgery Department, Dupuytren University Hospital, Limoges, France; 3 Cytology and Histology Department, Dupuytren University Hospital, Limoges, France; Morgagni-Pierantoni Hospital, ITALY

## Abstract

Pressurized intraperitoneal aerosol chemotherapy (PIPAC) is a valuable therapeutic alternative for patients with peritoneal metastases. PIPAC uses a hyaluronic acid-based gel to reduce surgically induced adhesions. The aim of this study was to evaluate the effects of the hyaluronic acid-based gel on tumor dissemination. First, we explored whether the survival of CT26 luciferase-expressing murine colonic tumor cells was correlated with the dose of HyaRegen® Gel, and we determined the half-maximal inhibitory concentration (the IC50) of the gel. Next, we performed an in vitro study of cell survival rates after gel application on day 0 (D0) and day 1 (D1). Finally, we intraperitoneally administered the gel to mice with immunocompetent BALB/c colonic peritoneal metastases (on D0, D5, D10, D14, and D18). Tumor growth was regularly monitored using a bioluminescence assay (on D11, D17, and D21). After all mice had been sacrificed on D21, the body weights and the volumes of intraperitoneal ascites were measured; the Peritoneal Carcinosis Index (PCI) and Ki-antigen 67 scores were calculated. The IC50 value was 70 μL of gel in a total volume of 100 μL. The cell survival rates on D4 were identical in the control group and the two groups that had been treated with gel on D0 and D1. The bioluminescence levels over time were similar in the gel and control groups. The PCI scores were 35.5 ± 2.89 for the control group and 36 ± 2.45 for the gel group (p = 0.8005). The mean Ki-67 index percentages were 37.28 ±1 1.75 for the control group and 34.03 ± 8.62 for the gel group (p = 0.1971). This in vitro and in vivo study using a mouse model of immunocompetent metastatic peritoneal cancer did not reveal any pro- or anti-tumoral effect of HyaRegen® Gel. These findings indicate that the gel can be used to treat PIPACs with minimal apprehension.

## Introduction

Colorectal cancer is associated with abnormally rapid and uncontrolled cell proliferation. Peritoneal metastases (PMs) develop in 10% to 25% of patients with colorectal cancers [1]. One innovative concept enhances the efficacy of intraperitoneal (IP) chemotherapy used to treat PMs by exploiting the physical properties and pressures of gases.

Pressurized intraperitoneal aerosol chemotherapy (PIPAC) was recently identified as an ingenious and useful IP drug delivery method in several experimental and clinical studies [2]. This method was developed to overcome the pharmacological limitations of conventional IP administration, including poor drug distribution within the abdominal cavity. PIPAC is an invasive procedure; repetitive laparoscopy is required every 6 weeks. This laparoscopy approach commonly triggers adhesion formation, creating difficulties during later access to the abdominal cavity. The adhesions are attributable to the formation of contact areas, exhibiting fibrous tissue, between adjacent internal organs. Adhesions hinder surgeons during dissection and make the abdominal approach difficult during later PIPACs. To prevent formation of such adhesions, some surgeons use tools that limit the development of tumor-adjacent tissue.

Hyaluronic acid (HA) is a natural disaccharide polymer composed of D-glucuronic acid and D-N-acetylglucosamine monomers linked by alternating β-1,4 and β-1,3 glycosidic bonds [3]. HA is present in large quantities in the human body; more than half of all HA is located in the skin, particularly in the dermis. HyaRegen® Gel (BioRegen Laboratory) is a sterile, transparent viscous gel; its active component is cross-linked HA molecules. The gel creates a barrier preventing tissue adhesion during healing after surgery.

The aim of this experimental study was to evaluate *in vitro* and *in vivo* the effects of HyaRegen® Gel on respectively CT26 cells and colorectal PM proliferation. We evaluated cellular viability and tumor proliferation after gel application.

## Materials and methods

### 1) Cell culture

The CT26-luc tumor line with the gene encoding firefly luciferase was derived from a BALB/c mouse colorectal adenocarcinoma cell line (Creative Biogene, France). It was then cultured in RPMI 1640 medium with 10% fetal calf serum and 2 mM L-glutamine (Dominique Dutscher, Bernolsheim, France), 0.2% glucose, 100 U/mL penicillin, and 100 μg/mL streptomycin (Thermo-Fisher Scientific, Illkirch, France), at 37°C with 5% CO2 in an atmosphere of 95% relative humidity.

### 2) In vitro study: The effect of the gel on cell proliferation

#### a) Median inhibitory concentration (IC) (IC50)

Eight groups (n = 6 wells each with 30,000 CT26 cells expressing luciferase) were established using different concentrations of HyaRegen® gel (0.5%, 1%, 2%, 4%, 8%, 15%, 25%, and 50%), along with a control group (0%).

#### b) Cell survival after HyaRegen® gel application on D0 and D1

Three groups (n = 2 wells each with 30,000 CT26 cells expressing luciferase) were analyzed:

- 2% of HyaRegen® gel in 2 mL on D0 (immediately after seeding),
- 2% of HyaRegen® gel in 2 mL NaCl on D1 after cell seeding,
- control group (0% of HyaRegen® gel).

#### c) Counting of cells on D4 via flow cytometry after seeding

Each cell pellet was resuspended in 1 mL of RPMI medium to obtain the total number of cells, as well as the percentage of living cells using a flow cytometer (Count and Viability Kit, Muse® Cell Analyzer, Millipore).

### 3) In vivo study: Tumor proliferation after HyaRegen® Gel application

#### a) Orthotopic syngeneic transplantation of CT26-luc cells

The CT26-luc cells (n = 30.000 cells) were injected IP into 8 BALB/c immunocompetent female mice (4-week-old, cAnNRj, Janvier-Labs, Le Genest-Saint-Isle, France). Animals were housed in 2 cages (2 test mice and 2 control mice in each cage).

Two groups of 4 mice (8 mice total) were established:

- The test group received an injection of the gel solution (40 μL HyaRegen® in 100 μL 0.9% saline solution) at 2 h after the injection of CT26-luc cells.
- The control group received an injection of 100 μL 0.9% saline solution, at 2 h after the injection of CT26-luc cells.

The injections were repeated on D5, D10, D14, and D18.

#### b) Bioluminescence imaging

Bioluminescence imaging was performed on D11, D17, and D21 after CT26-luc transplantation. Mice under isoflurane anesthesia were injected with 200 μL of 150 mg/kg Xeno-Light D-Luciferin (PerkinElmer, Waltham, MA, USA) into the peritoneal cavity and imaged using a cooled charge-coupled device camera system at 5 min after injection (IVIS Lumina System Series III, PerkinElmer). The total bioluminescent signal intensity in the abdominal region was calculated using Living Image 4.0 software (PerkinElmer) in both groups of mice (n = 4 gel/ n = 4 control). All mice that have developed peritoneal metastasis were included for further experiments.

#### c) Weight, PCI score, and ascites analysis

At 21 days, all mice were weighed, sacrificed by CO2 inhalation, and immediately subjected to complete midline laparotomy. The abdominal cavity was inspected, and the PCI score was calculated [4, 5]. A volume measurement of ascites (median) and a macroscopic analysis were performed concurrently (n = 4 for each group).

#### d) Proliferating cells and histologic response

24 PMs were collected from 8 mice, fixed in 4% paraformaldehyde and embedded in paraffin. Sections (4-μm-thick) were cut and stained with Hematoxylin-Eosin-Safran (HES) and Ki-67 antigen probes (anti-Ki-67 Rabbit Monoclonal Primary Antibody Ventana 790–4286, Roche) using an automated Benchmarck XT instrument (Roche) as previously described [6]. A surface index based on Ki-67 status was established to compare proliferation rates (%) in n = 34 for gel group and n = 35 for control group with Fiji/ImageJ (NIH). Histological response (HR) score is obtained by visual assessment in 3 nodules per mouse [7].

### 4) Ethics

All mice were housed in ERET cages (University Limoges, France), and provided with aspen wood bedding (Lab Mix; Serlab, France), refuge mouse huts (Serlab) and cocoon products (Serlab) for environmental enrichment. Tap water and food pellets (RM1 Entretien, France) were provided ad libitum. The animal room was maintained under controlled temperature (21°C), photoperiod (reversed 12/12 h light/dark cycle: lights on between 19:00–07:00h) and relative humidity (50–60%) conditions, monitored by an automatic controller. All animals were checked daily, with cages changed twice per week.

Analysis of the welfare score (score from 0 to 12) included: mouse fur appearance, behavior, wounds, weight loss, tumor development (2 cm maximum), fecal appearance. If this score was higher than 8, the mouse was euthanized immediately.

All the researchers received special training in animal care and handling before starting the study.

All experiments were approved by the French Ministry of Research (APAFIS # 1 1942–2017102611003706) under the hand of S Bardet, approved by the local Ethics and Animal Protection Committee (decree n° 2001–131, European directive 86-609-CEE 1986). All animal care and experimental procedures were conducted in accordance with French legislation of 2013 and the European Community directives (Directive 2010/63/EU on the care and use of laboratory animals).

### 5) Statistical analysis and graphics

GraphPad Prism software was used for all statistical analyses using means and standard deviation. P<0.05 was considered statistically significant. Images and graphics were drawn with either GraphPad Prism software or Microsoft PowerPoint. All experiments were conducted in a blind fashion.

## Results

### 1) Median inhibitory concentration (IC) (IC50) and cell survival at D4 after in vitro gel application on D0

**Fig 1:**
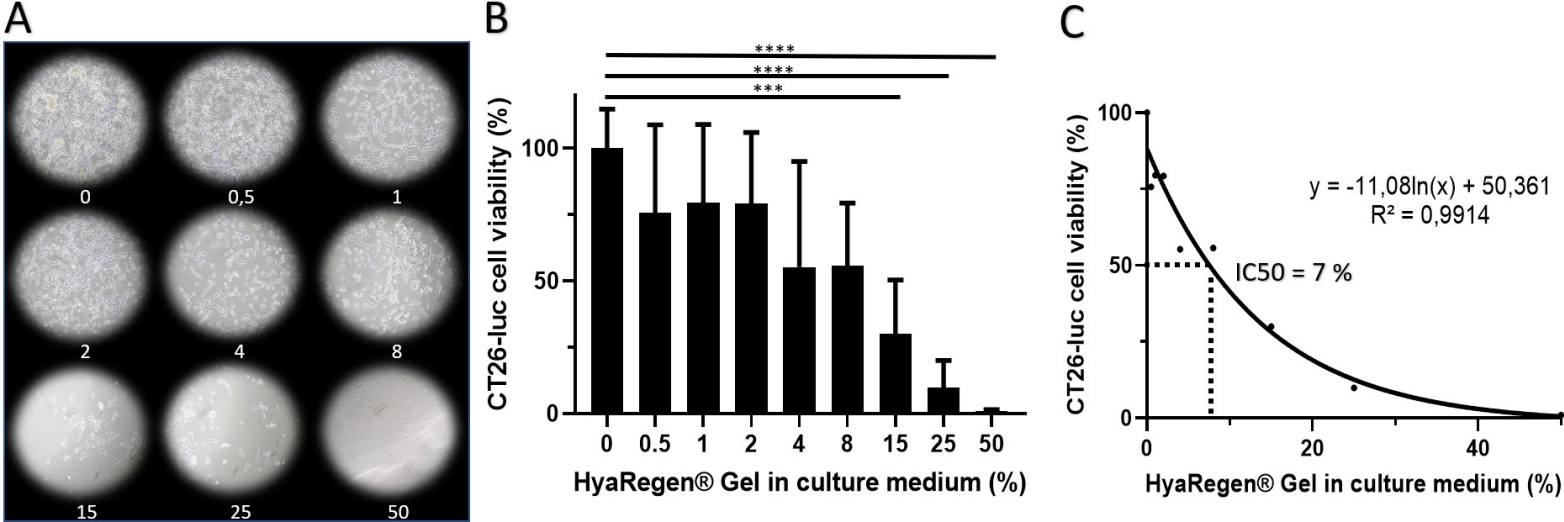
CT26- luc cell viability is affected by increasing amount of gel on D4 after seeding. A photograph of the cell cultures on D4 according to gel percentages (A). The percentages of viable cells on D4. Mean +-SD. * for p value <0.05, ** <0.01, *** <0.001 and **** <0.0001. (B). Logarithmic regression curve between gel concentration and viability indicates an IC50 around 7% of HayRegen® gel (C).

In order to precise a potential toxic effect of the HyaRegen® gel, we have measured in vitro CT26-luc viability by flow cytometry with different concentration of gel (from 0 to 50%, Fig 1A). There was a progressive decrease in the number of viable CT26-luc cells as a function of the gel concentration applied. A one-way analysis of variance (ANOVA) with Tukey test for multiple comparison confirmed a significative effect on cell viability at high gel concentration (F (8, 45) = 11,21 P<0,0001) (**Fig 1B and Table 1**). In **Fig 1C**, the IC50 dose was estimated approximately at 7% of gel.

**Table 1:** Statistic tests for in vitro cell viability regarding gel concentration. A one-way analysis of variance (ANOVA) with Tukey test for multiple comparison confirmed a significative effect on cell viability at high concentration of HyaRegen® gel (F (8, 45) = 11,21 P<0,0001).

0 vs. 15	70,05	23,34 to 116,8	***	0,0004
0 vs. 25	90,12	43,42 to 136,8	****	<0,0001
0 vs. 50	99,01	52,31 to 145,7	****	<0,0001
0.5 vs. 25	65,84	19,14 to 112,5	**	0,0011
0.5 vs. 50	74,73	28,03 to 121,4	***	0,0001
1 vs. 15	49,56	2,857 to 96,27	*	0,0300
1 vs. 25	69,63	22,93 to 116,3	***	0,0005
1 vs. 50	78,52	31,82 to 125,2	****	<0,0001
2 vs. 15	49,32	2,611 to 96,02	*	0,0314
2 vs. 25	69,39	22,68 to 116,1	***	0,0005
2 vs. 50	78,28	31,57 to 125,0	****	<0,0001
4 vs. 50	54,28	7,576 to 101,0	*	0,0122
8 vs. 50	54,75	8,047 to 101,5	*	0,0111

### 2) Tumor proliferation after HyaRegen® Gel application

For *in vivo* experiments, the HyaRegen® dose was calculated according to the animal body surface. In human, the HyaRegen® dose is 100 mL for 1.7 m^2^. In mice, the body surface is about 36 cm^2^ for 40 μL of IP-injected gel.

#### a) Bioluminescence imaging and quantification of tumour growth

**Fig 2:**
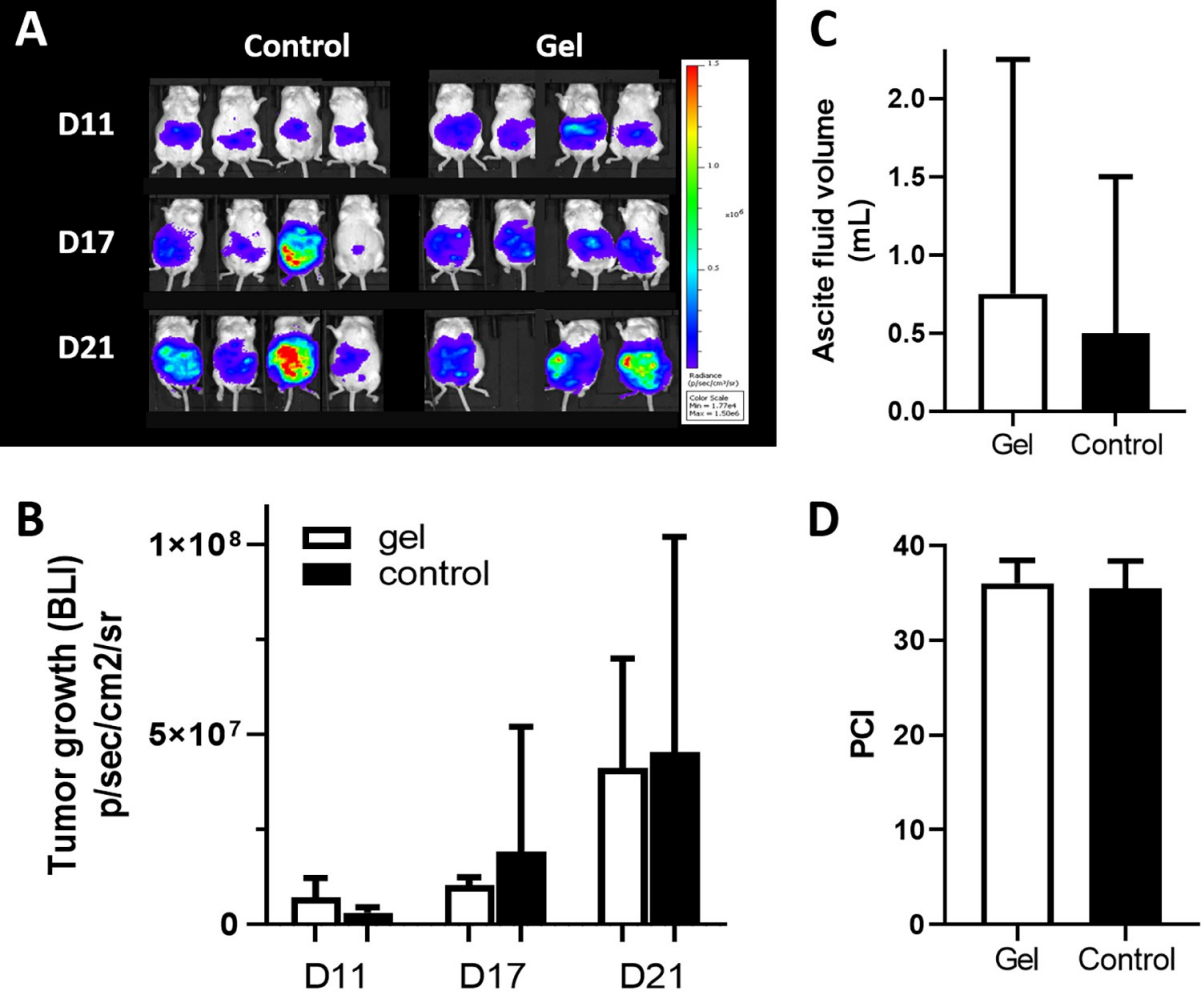
In vivo analysis of HyaRegen® Gel effects on tumor proliferation, dissemination and ascites. In vivo quantification of tumor growth via bioluminescence tracking of luciferase-expressing CT26 cells on days 11, 17, and 21 after gel administration: BLI mice photography (A) and tumor growth histograms, mean +-SD,* for p value <0.05 (B). Comparison of ascites volume between the control and gel groups on D21, mean +-SD (C). Comparison of median peritoneal cancer index (PCI) between the control and gel groups (D).

An increase in the bioluminescence signal (BLI) over time is noted in both groups (**Fig 2A and 2B**) as previously described by our team [8] and is statistically significant (mixed effects model and multiple comparison Sidak test, p = 0.029, F(2,11) = 4.99). The mean BLI indices were 3.05.10^6^ ± 1.44.10^6^ for the control group versus 7.18.10^6^ ± 5.06.10^6^ for the gel group on D11; 1.92.10^7^ ± 3.27.10^7^ and 1.03.10^7^ ± 2.14.10^6^ on D17; and 4.54.10^7^ ± 5.66.10^7^ and 4.12.10^7^ ± 2.87.10^7^ on D21. However, no significant difference in tumor growth was observed between the 2 groups (control versus gel, p = 0.853, F(1,6) = 0.0372).

#### b) Weight, ascites and PCI

During the 21-day period, one mouse had a welfare score greater than 8 and was euthanised at D19. At the end of the experiment (day 21), the mean body weights were 19.99 ± 0.48 g in the control group and 21.16 ± 1.52 g in the gel group (p = 0.34). There was no significant difference between the two groups. The control mice exhibited a mean hemorrhagic ascites volume of 0.5 ± 1 mL versus 0.75 ± 1.5 mL in the gel group (p = 0.7908, unpaired t test) (**Fig 2C**). The PCI scores were 35.5 ± 2.89 for the control group and 36 ± 2.45 for the gel group (p = 0.8005 unpaired t test) (**Fig 2D**). The BLI after euthanasia was not statistically different between both groups (p = 0.9135 unpaired t test, **Fig 2B**).

### 3) Determination of the percentage of surface marked with Ki-67

**Fig 3:**
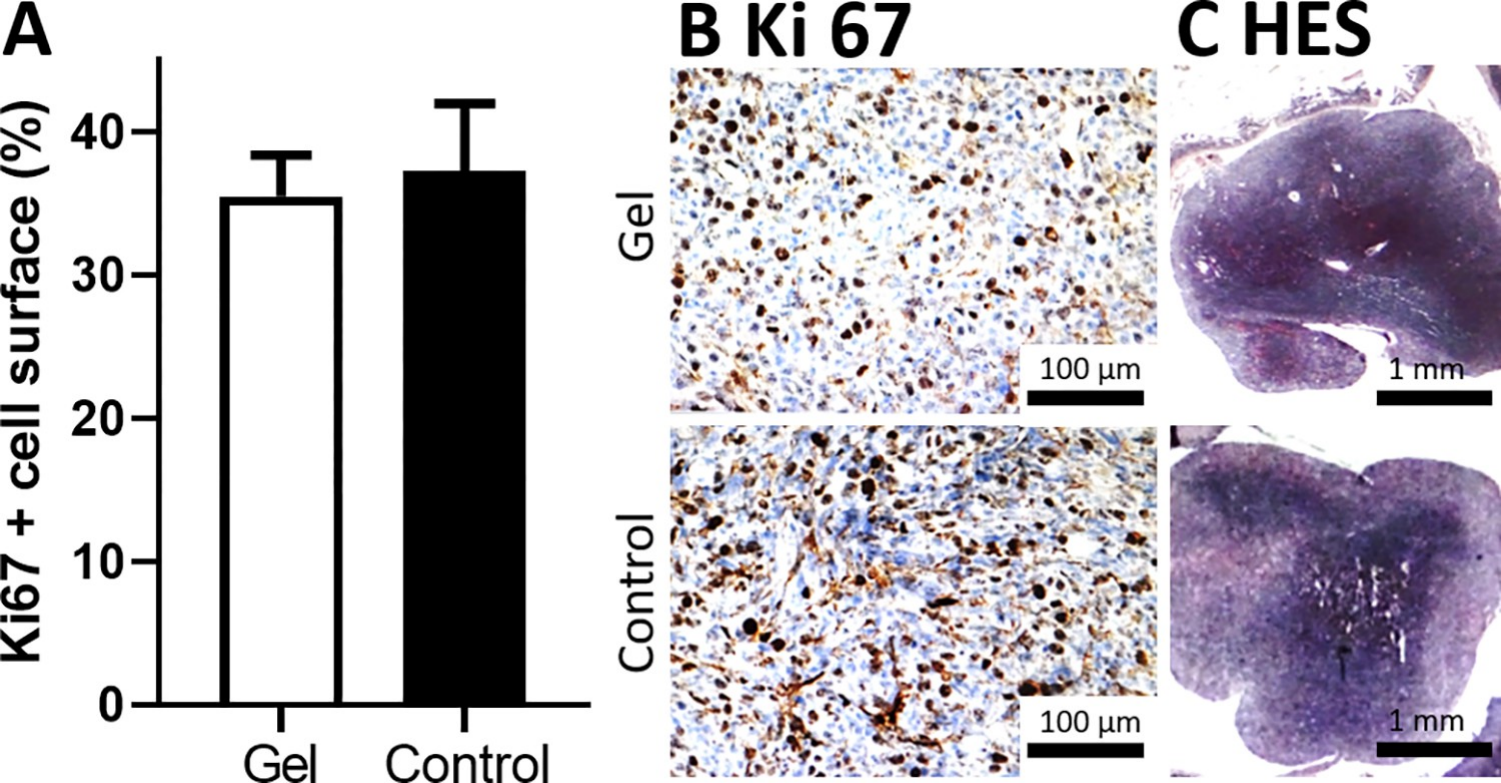
Histograms showing in vivo proliferating cells (Ki-67 antigen labeling) for the control and gel groups, mean +-SD (A). Micrography showing Ki-67 (B) and HES staining (C).

Biopsies during exploration allow in-depth analysis of cancerous tissue through immunohistochemical methods, specifically detection of a 360 kDa nuclear protein KI-67 which is present in proliferating cells [9]. The mean Ki-67 percentages were 37.28 ± 11.75% for the control group and 34.03 ± 8.62% for the gel group (**Fig 3A and 3B**). There was no significant difference between the two groups (unpaired t test p = 0.1971). Classical staining (Hematoxylin-eosin saffron HES) shows similar structure in gel treated and control tumors with no histological response (**Fig 3C**).

S1 File

## Discussion

Hyaluronic gel is often used during surgery and after PIPACs to avoid postoperative adhesions, but the literature suggests that this usage can increase tumor dissemination. Our in vitro and in vivo experiments did not reveal either a positive or negative effect of hyaluronic gel on cell proliferation or tumor proliferation in mice with colorectal PMs.

HA is the principal glycosaminoglycan in the extracellular matrix. HA is suspected to regulate the tumor microenvironment by interacting with specific receptors and transducing intracellular signals that promote the malignant phenotype. Consequently, high levels of HA have been identified in various cancers (such as breast, prostate, lung, and ovarian cancers) [10, 11]. Additionally, several clinical studies have shown that HA promotes invasion, motility, and epithelial-to-mesenchymal transition in breast cancer cells by triggering various signaling pathways and upregulating the expression of collagen-degrading enzymes [12, 13]. There is increasing evidence of a relationship between tumor expression of HA and poor outcomes among patients with ovarian, gastric, and breast carcinomas [14, 15]. Llaneza *et al*. analyzed the impact of HA on colorectal cancer and concluded that elevated tumor cytosolic HA levels significantly reduced relapse-free survival and overall survival [16]. An antitumour effect was observed in our *in vitro* experiments, with a statistically significant reduction of viable tumour cells following application with high gel concentration. However, the increased viscosity of the gel may have caused cell asphyxia and contributed to cell death.

In our study using a mouse model of PCI, we found that the BLI rate increased over time in both groups at the same rate, and we confirmed our previously published observations of tumor proliferation after IP injection of CT26-luc cells [8]. However, there was no difference in BLI rate between gel and control groups. This lack of difference was confirmed during laparotomy (the PCI rate was identical in both groups).

However, contrasting results have been reported in some studies. Two studies exploring the use of HyaRegen® gel in models of PMs demonstrated that the gel inhibited migration, invasion, and proliferation of cancer cells in vitro, as well as the implantation of such cells in animal models, indicating that HA could potentially serve as an anti-tumor agent [17, 18].

In the study by Pang et al. [17], the experimental protocol was similar to that of our work, but our gel exposure time was longer. Those authors showed that the gel reduced the invasion of ovarian cancer cells in an immunocompromised mouse model, specifically through the inhibition of signals initiated by epidermal growth factor and the reduced expression of proteins linked to metastasis (such as Rac1 protein).

A second study on gastric and liver PMs was performed by Lan et al. [18] over 7 weeks (i.e., longer than our study). Those authors also found that the gel inhibited signals mediated by epidermal growth factor, and they characterized the expression patterns of proteins involved in the metastatic process. As demonstrated in the study on PMs of ovarian origin, it is possible that the positive results arose from the absence of immune defenses, the lack of interaction between the tumor and the microenvironment, and the longer exposure to the gel.

Immunity plays a key role in pro- and anti-tumor cell signaling [19], facilitating immunotherapy for certain patients [20]. This is why we chose to perform our experiments on immunocompetent BALB/c mice; we sought to preserve the interaction between the microenvironment and tumor cells. Moreover, previous studies used the HA molecule alone, rather than an HA-based gel. The reticular conformation of HyaRegen®, as well as its unique composition, imparted distinct properties to the gel, thus explaining the different results.

Further research is needed to thoroughly evaluate the prognostic utility of HA. The techniques used vary among teams; some analyze HA levels in plasma, whereas others sample tumor tissues or stroma. Additionally, the lack of a gold standard for detection of an HA threshold hinders interpretation of the results. Finally, although HA use is common in current practice, the quantity of gel in the peritoneal cavity during the PIPAC procedure is low.

## Conclusion

This in vitro and in vivo study using a mouse model of immunocompetent metastatic peritoneal cancer did not reveal any pro-tumoral or anti-tumoral effects of HyaRegen® Gel. This result encourages gel use during PIPAC, with minimal apprehension.

## Supporting information

S1 Checklist (DOCX)

S1 File (DOCX)
