# Supplementary material for: Effects of HyaRegen gel on tumour proliferation of colorectal peritoneal metastases
Source: PLoS One. 2024 Sep 10;19(9):e0307965. doi: 10.1371/journal.pone.0307965 (PMC11386418; doi:10.1371/journal.pone.0307965)

FIG1


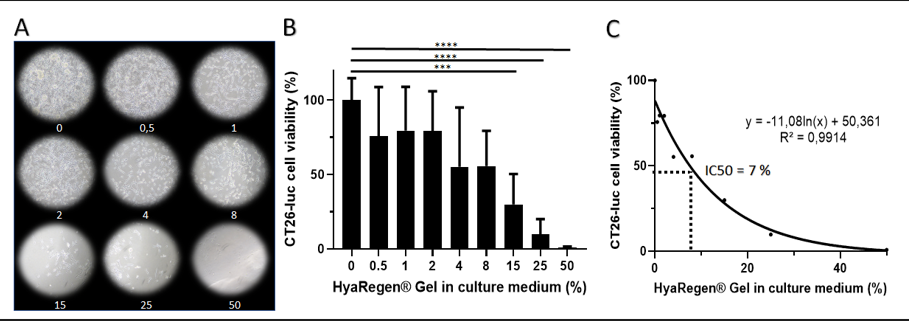


B

| 0 | 0.5 | 1 | 2 | 4 | 8 | 15 | 25 | 50 |
| --- | --- | --- | --- | --- | --- | --- | --- | --- |
| 86,4729446 | 82,70749438 | 48,79049062 | 27,53728829 | 13,25393122 | 22,76656822 | 29,30121922 | 26,03879941 | 1,017401001 |
| 106,1934125 | 78,05569896 | 43,47381216 | 82,11619252 | 34,64205845 | 70,95930142 | 9,987734708 | 1,828844534 | 1,258364358 |
| 105,9223805 | 88,62730325 | 119,5962852 | 84,61379112 | 82,65147577 | 69,77092873 | 29,61325966 | 16,97573527 | 1,552875288 |
| 97,66412962 | 92,96847689 | 85,32807635 | 91,52310084 | 12,2679648 | 28,07767641 | 47,10576953 | 0,595714887 | 1,512714728 |
| 81,57865943 | 10,21592726 | 79,35661736 | 84,4026378 | 99,65925991 | 70,34862669 | 58,11426875 | 11,0581997 | 0,267736904 |
| 122,1684733 | 101,759551 | 100,5290686 | 105,4036697 | 89,14505076 | 72,52392625 | 5,582410182 | 2,777818078 | 0,327977983 |

| Number of values | 6 | 6 | 6 | 6 | 6 | 6 | 6 | 6 | 6 |
| --- | --- | --- | --- | --- | --- | --- | --- | --- | --- |
|  |  |  |  |  |  |  |  |  |  |
| Mean | 100,0 | 75,72 | 79,51 | 79,27 | 55,27 | 55,74 | 29,95 | 9,879 | 0,9895 |
| Std. Deviation | 14,78 | 33,13 | 29,41 | 26,73 | 39,77 | 23,56 | 20,40 | 10,14 | 0,5697 |
| Std. Error of Mean | 6,035 | 13,52 | 12,01 | 10,91 | 16,24 | 9,620 | 8,327 | 4,140 | 0,2326 |

C

| 0 | 100 |
| --- | --- |
| 0,5 | 75,72241 |
| 1 | 79,51239 |
| 2 | 79,26611 |
| 4 | 55,26996 |
| 8 | 55,74117 |
| 15 | 29,95078 |
| 25 | 9,879185 |
| 50 | 0,989512 |

FIG 2


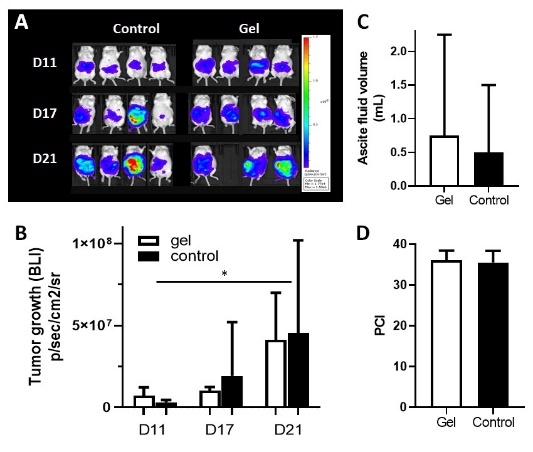


B

| Days | Control | | | | Gel | | | | | |  |
| --- | --- | --- | --- | --- | --- | --- | --- | --- | --- | --- | --- |
| 11 | 5200000 | 2580000 | 2200000 | 2250000 |  | 6950000 | 3240000 | 1,44e+007 | 4140000 |  | |
| 17 | 7730000 | 646000 | 6,81e+007 | 507000 |  | 1,02e+007 | 1,32e+007 | 8080000 | 9740000 |  | |
| 21 | 3,97e+007 | 1,07e+007 | 1,27e+008 | 4090000 |  | 1,76e+007 |  | 3,29e+007 | 7,32e+007 |  | |


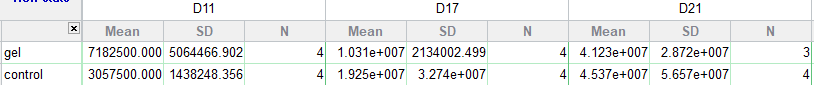


C D


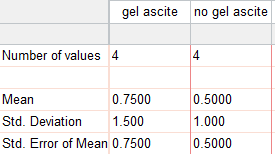

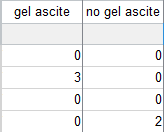

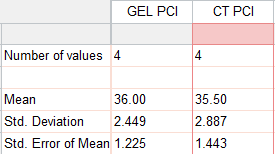

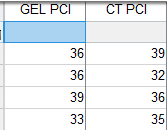


FIG3


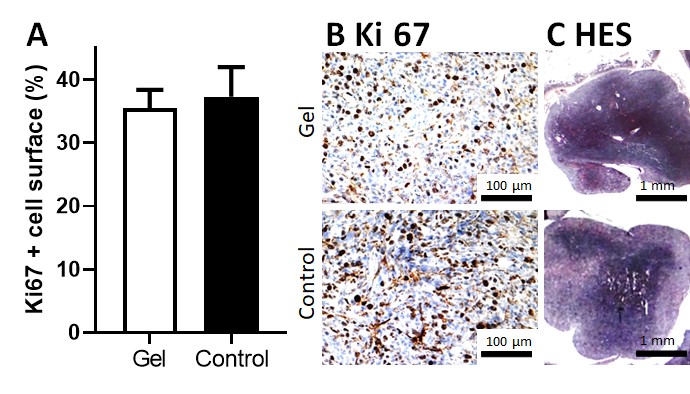


A


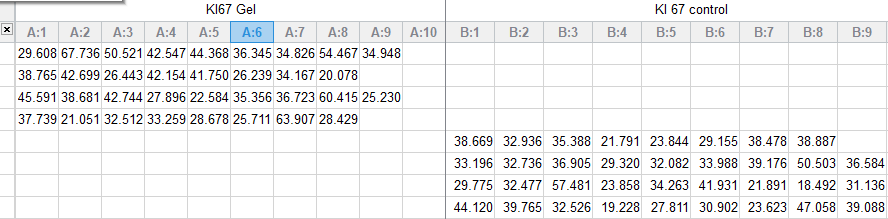


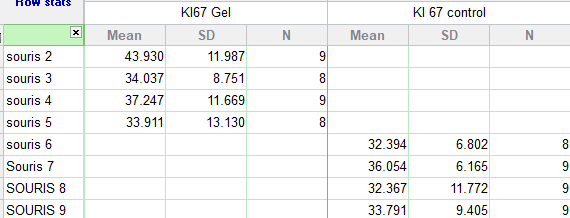

Supplement: S1 File — (DOCX) [file pone.0307965.s002.docx]
